# Sterile Pyuria in Kawasaki Disease: A Large Prospective Cohort Study

**DOI:** 10.3389/fcvm.2022.856144

**Published:** 2022-05-11

**Authors:** Xiaoliang Liu, Lin Wang, Shuran Shao, Nanjun Zhang, Mei Wu, Lei Liu, Yimin Hua, Kaiyu Zhou, Li Yu, Hua Wang, Chuan Wang

**Affiliations:** ^1^Department of Pediatric Cardiology, West China Second University Hospital, Sichuan University, Chengdu, China; ^2^Key Laboratory of Birth Defects and Related Diseases of Women and Children, Sichuan University, Ministry of Education Chengdu, Chengdu, China; ^3^Key Laboratory of Development and Diseases of Women and Children of Sichuan Province, West China Second University Hospital, Sichuan University, Chengdu, China; ^4^Longquanyi District of Chengdu Maternity & Child Health Care Hospital, Chengdu, China; ^5^West China Medical School of Sichuan University, Chengdu, China; ^6^Department of Pediatrics, West China Second University Hospital, Sichuan University, Chengdu, China; ^7^The Cardiac Development and Early Intervention Unit, West China Institute of Women and Children's Health, West China Second University Hospital, Sichuan University, Chengdu, China

**Keywords:** sterile pyuria, Kawasaki disease, intravenous immunoglobulin resistance, vasculitis, coronary artery lesions

## Abstract

**Background:**

Kawasaki disease (KD) is an acute systemic vasculitis and is becoming the leading cause of acquired cardiac disease in Children. Sterile pyuria is a known complication of KD. However, its associations with the inflammatory reaction severity, IVIG resistance as well as coronary artery lesions (CALs) in KD remain elusive.

**Aims:**

We aimed to analyze the clinical profiles of sterile pyuria in KD, to determine whether sterile pyuria is an indicator of the disease severity in patients with KD, and to assess the associations between sterile pyuria and IVIG resistance as well as CALs.

**Methods:**

We prospectively collected data from 702 patients with KD between January 2015 and June 2020. Profiles of patients with sterile pyuria (group A, *n* = 63) were compared to those of patients without sterile pyuria (group B, *n* = 639). The associations between sterile pyuria and IVIG resistance as well as CALs in KD were further determined by univariate and/or multivariate logistic regression analysis.

**Results:**

Sterile pyuria was observed in 9.0% of patients with KD, without predominance in age spectrum and gender. The levels of neutrophil percentages, alanine transaminase, total bilirubin, blood urea nitrogen, creatinine, the incidence of initial IVIG resistance, and rate of moderate/giant coronary artery aneurysms (CAAs) were significantly higher in group A than that in group B. Sterile pyuria was identified as an independent risk factor for initial IVIG resistance, yielding high specificity (92.7%) and low sensitivity (18.5%). However, sterile pyuria was not associated with repeated IVIG resistance and persistence of CALs in KD.

**Conclusion:**

The incidence of sterile pyuria is relatively low in KD patients. Patients with sterile pyuria in KD exhibited a more severe inflammatory burden and were more likely to develop the initial IVIG resistance and moderate/giant CAAs. The overall prognosis of KD patients with sterile pyuria was satisfactory.

## Introduction

Kawasaki disease (KD) is an acute systemic vasculitis and is becoming the leading cause of acquired heart disease in children worldwide (1, 2). The prevalence of KD per 100,000 children <5 years old is various by race: 309.0–330.2 in Japan, 71.9–110.0 in China, 170.9–194.9 in Korea, 18.1–21.3 in the USA, and 49.4 in Hawaii. The rate of KD in children from East Asia is 10–15 times that in Caucasian children (3, 4). Apart from the classical clinical features, multiple organs and tissues involvement, such as the pulmonary, musculoskeletal, gastrointestinal, neurological, and urinary systems, have been well recognized and reported in patients with KD (1). Sterile pyuria has been recognized as the most common manifestation of the renal complications associated with KD (5).

However, owing to the small sample size, retrospective study design, and unstandardized or even absent diagnostic criteria, the incidence of sterile pyuria in KD among different studies was inconclusive and was reported to range from 30 to 80% (5–9). The association between sterile pyuria and the inflammatory reaction severity in KD was only preliminarily explored in two pilot studies (6, 7). Watanabe et al. from Japan in 2007 (6) and Choi et al. from Korea in 2013 (7) described the clinical characteristics of KD patients with sterile pyuria and suggested that these children exhibited more severe inflammatory reactions and may have sub-clinical renal injuries. However, these two studies were limited by the small sample size (*n* = 23 and *n* = 133, respectively) as well as the retrospective nature of the study design. It remains to be further verified in a prospective study with a large sample size.

Timely intravenous immunoglobulin (IVIG) treatment is quite effective; however, approximately 10–20% of patients with KD are resistant to IVIG treatment and at an increased risk of developing CALs (10). Therefore, the prediction of IVIG resistance and CALs are the pivotal topic of interest in KD since these patients may benefit from adjunctive therapies for primary treatment, including corticosteroids, infliximab, cytotoxic agents, and plasma exchange (1, 11). However, data with respect to the predictive value of sterile pyuria in IVIG resistance and the occurrence or persistence of CALs in patients with KD are lacking.

Therefore, the present study aimed (1) to systematically and prospectively elaborate on the clinical features of KD patients with sterile pyuria; (2) to assess whether the presence of sterile pyuria reflects the severity of KD, and (3) to evaluate the predictive ability of sterile pyuria in IVIG resistance and CALs in patients with KD.

## Methods

Patients with KD were prospectively recruited from January 2015 to June 2020 at West China Second University Hospital of Sichuan University (WCSUH-SCU), which is the largest medical center for children in Southwest China. WCSUH-SCU has 730 registered beds, 16 clinical departments and divisions. Structured questionnaires with pre-coded questions that included basic demographic information, clinical presentations, laboratory examination results, treatment, and follow-up outcomes, were used for the data collection. Informed written consent was obtained from the parents' guardians. The Ethics Committee approved the study on Human Subjects at Sichuan University.

The inclusion criteria were: (1) age ≤ 18 years old; (2) meeting the diagnostic criteria for complete or incomplete KD in accordance with the 2004 American Heart Association (AHA) recommendations for KD (2); (3) receiving a standardized treatment regimen within 10 days from fever onset at our hospital; (4) blood sampling and two consecutive urine tests were conducted before initial IVIG infusion; (5) negative urine culture. The exclusion criteria were: (1) incomplete hematological data; (2) having other causes of sterile pyuria before KD onset, including renal tuberculosis, renal abscess, renal and urinary tract anomalies, systemic lupus erythematosus, nephrolithiasis, interstitial nephritis, glomerulonephritis, interstitial cystitis, and inflammation near the ureter or bladder et al.; (3) incomplete follow-up data.

Clean, voided, and mid-stream urine was collected from the toilet-trained children, whereas if children did not cooperate with urine collection, urine samples were collected using a sterile urine collecting bag over the perineum after skin disinfection. Well-trained nurses or doctors conducted urine collection. Pyuria was defined as five or more white blood cells (WBCs)/high-power field (HPF) on urine microscopy for two consecutive urine tests. The severity of pyuria was further classified as mild (5–20 WBCs/HPF), moderate (21–30 WBCs/HPF), and severe (>30 WBCs/HPF). Microscopic hematuria [five or more red blood cells (RBCs)/HPF on urine microscopy] and pyocytes were also recorded. A urine culture was performed for all patients with pyuria. Sterile pyuria was defined as pyuria with a negative urine culture (2). All the included patients with sterile pyuria had normal urine test results after IVIG infusion.

All patients with KD underwent the same treatment program after the diagnosis of KD established (2). IVIG (2 g/kg) and aspirin (30–50 mg/kg/day) were administered during the acute phase of KD. After the resolution of a patient's fever, the dose of aspirin decreased to 3–5 mg/kg/day and continued for 6–8 weeks. In patients with CALs, aspirin continued until the patients exhibited no sign of any change that had occurred in the coronary arteries. Patients with recurrent or persistent fever for ≥36 h after the first IVIG administration were treated with a second course of IVIG (2 g/kg). The initial IVIG resistance was defined as persistent or recurrent fever (oral temperature ≥ 38.0 °C) for more than 36 h but less than 7 d after the first IVIG infusion (12). Furthermore, if the patient had recurrent or persistent fever, even after the second IVIG infusion, which was defined as repeated IVIG resistance; adjunctive therapy constituting the tapered administration of pulse intravenous methylprednisolone (20–30 mg/kg/day for three consecutive days) followed by oral prednisone (2 mg/kg/day) for 7 d was employed. CALs were defined on the normalization of dimensions for body surface area (BSA) as Z scores according to the AHA scientific statement of KD (1, 2). Based on Z scores, the CALs were further classified as follows: dilation: 2.0 to <2.5; small aneurysm: ≥2.5 to <5.0; medium aneurysm: ≥5.0 to <10.0, and absolute dimension <8 mm; giant aneurysm: ≥10, or absolute dimension ≥8 mm (1, 2). Patients who still had echocardiographic evidence of CAA after 1-year follow-up beyond KD onset were defined as CAA persistence since the subjects in the present study were recruited from January 2015 to June 2020. CAAs were considered to have regressed when the enlarged coronary arteries demonstrated a normal internal diameter without any irregularities at any time points during the 1-year follow-up duration.

**Figure 1:**
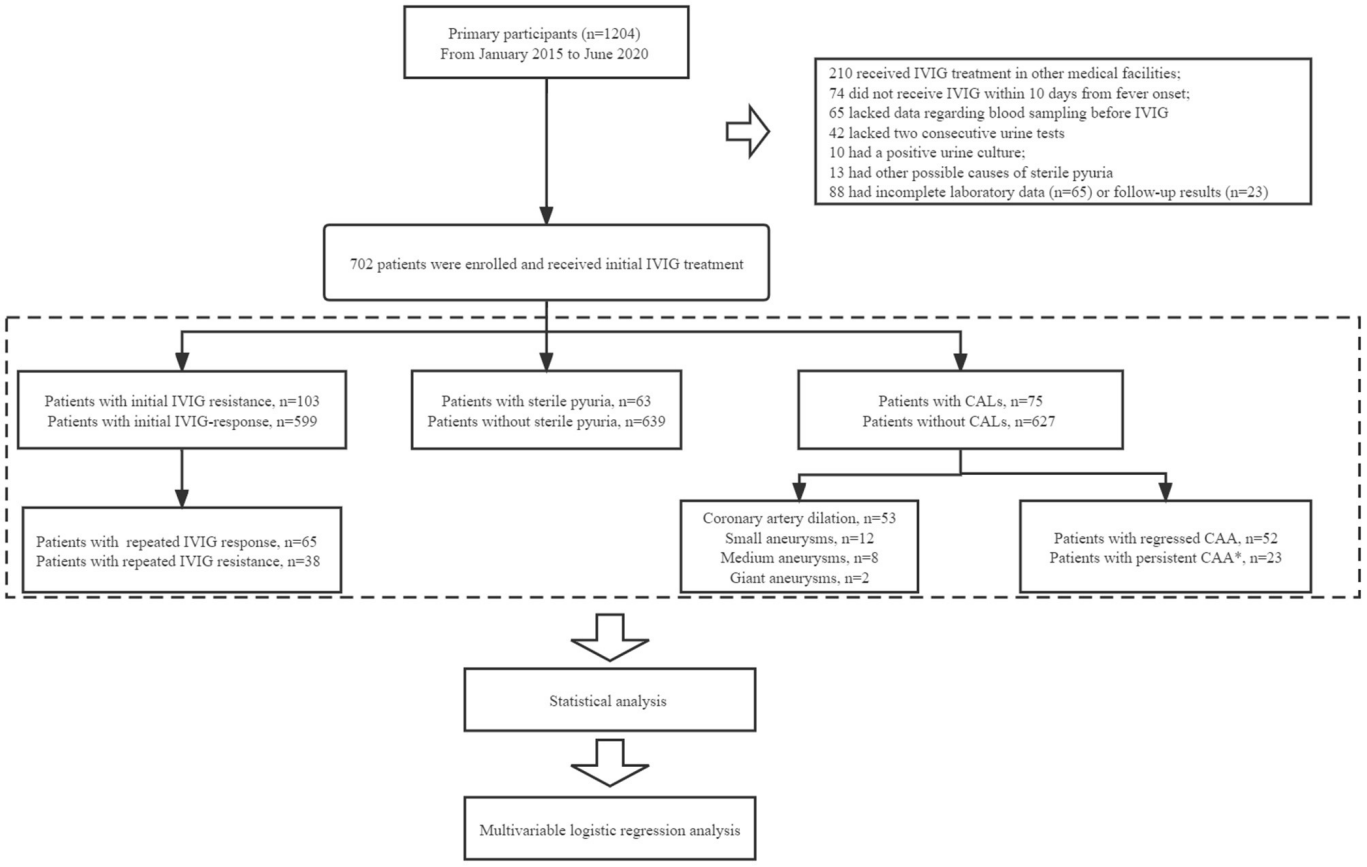
The flowchart of our prospective cohort study. In total, 1,204 patients were diagnosed with KD on admission. Patients who had received IVIG infusion at other medical facilities (*n* = 210), who did not receive IVIG treatment within 10 days after the onset of fever (*n* = 74), and who either did not undergo blood sampling (*n* = 65) or two consecutive urine tests (*n* = 42) before IVIG administration, were excluded. Patients with a positive urine culture (*n* = 10) or other possible causes of sterile pyuria (*n* = 13) were excluded. We excluded patients with incomplete laboratory data (*n* = 65) or follow-up results (*n* = 23). Finally, a total of 702 patients with KD were enrolled for analysis. All patients with KD were divided into different groups: IVIG resistance (*n* = 103), sterile pyuria (*n* = 63), CALs (*n* = 75). Of the 75 KD children with CALs, CAD, small CAAs, moderate CAAs, and giant CAAs were observed in 53, 12, 8, and 2 patients, respectively. Patients with CALs were further divided into the persistent CALs group (*n* = 23) and the regressed CALs group (*n* = 52). *Patients who still had echocardiographic evidence of CAA after 1-year follow-up beyond KD onset.

A total of 1,204 patients were diagnosed with KD on admission. Finally, 702 patients with KD were recruited into this study following the above inclusion criteria and exclusion criteria. These patients were divided into two groups, patients with sterile pyuria (group A, *n* = 63) and those without sterile pyuria (group B, *n* = 639). To assess the association between sterile pyuria and IVIG resistance, patients were divided into the initial IVIG resistance group (*n* = 103) and the initial IVIG response group (*n* = 599). Additionally, patients with initial IVIG resistance were further categorized into the repeated IVIG resistance group (*n* = 38) and repeated IVIG response group (*n* = 65). Of the 75 KD children with CALs, coronary artery dilation (CAD), small CAAs, moderate CAAs, and giant CAAs were observed in 53, 12, 8, and 2 patients, respectively. Given the small numbers of patients with moderate and giant CAAs, KD patients in the present study were divided into those without CALs, those with CAD/small CAAs, and those with moderate/giant CAAs. Based on the above definitions, patients with CALs were further divided into the persistent CALs group (*n* = 23) and the regressed CALs group (*n* = 52) (Figure 1).

### Statistical Analysis

Quantitative data are presented as the mean and range or mean ± standard deviation (SD), while the qualitative data are expressed as n/%. Shapiro–Wilk and homogeneity of variance tests were used to confirm that the quantitative data from the different groups exhibited a normal distribution with homogeneity of variance. Differences in the quantitative data from the two groups were assessed using the independent sample *t-*tests or the Mann–Whitney U-tests. To assess the non-parametric dichotomous and the non-dichotomous variables, we used the contingency tables/Fisher's exact tests and regression analyses with dummy variables, respectively. These crucial indicators from the univariate analysis were then subjected to the multivariate logistic regression analysis to identify the independent predictors. The level of statistical significance was set at *p* < 0.05 (two-tailed). All data were analyzed using SPSS version 21.0 (SPSS Inc. Chicago, IL, USA).

## Results

### Basic Clinical Characteristics of the KD Patients With Sterile Pyuria

Sterile pyuria was observed in 63 patients with KD (38 males, 25 females; M: F of 1.52:1), with an incidence of 9.0% (63/702). Of these patients, 11.1% (7/63) were younger than 6 months, 9.5% (6/63) were aged between 0.5 and 0.9 years, 19.0% (12/63) were aged between 1.0 and 1.9 years, 15.9% (10/63) were aged between 2.0 and 2.9 years, 15.9% (10/63) were aged between 3.0 and 3.9 years, 16.4% (9/63) were aged between 4.0 and 4.9 years and 14.3% (9/63) were older than 5 years. Mild, moderate, and severe sterile pyuria was observed in 25, 22, and 16 patients, respectively. Pyocytes in urine and microscopic hematuria were found in 28.6% (18/63) and 19.0% (12/63) of patients with sterile pyuria, respectively. For all patients with microscopic hematuria, the morphology of urinary RBCs was dysmorphic. The nitrite test was negative in all the patients with sterile pyuria. Acute kidney injury or failure and nephrotic syndrome were not observed in this cohort (Table 1).

**Table 1:** Clinical features of KD patients with sterile pyuria.

	**Patients, *n =* 63**
**Male/Female**	38/25 (1.52:1)
**Age distribution, years**	
0~0.5	11.1% (7/63)
0.5~0.9	9.5% (6/63)
1.0~1.9	19.0% (12/63)
2.0~2.9	15.9% (10/63)
3.0~3.9	15.9% (10/63)
4.0~4.9	16.4% (9/63)
5.0~	14.3% (9/63)
**Fever days**	
Total fever days (days)	12.6 (6.0–39.0)
Fever duration before IVIG (days)	11.8 (5.0–38.0)
Fever duration on admission (days)	10.2 (2.0–35.0)
**Complete KD/Incomplete KD**	69.8%/30.2% (44/19)
**IVIG resistance**	30.2% (19/63)
**CALs**	14.3% (9/63)
**Urine analysis**	
Sterile pyuria	
Mild, 5–20 WBCs/HPF	39.7% (25/63)
Moderate, 21–30 WBCs/sssHPF	34.9% (22/63)
Severe, >30 WBCs/HPF	25.4% (16/63)
Pyocytes	28.6% (18/63)
Microscopic hematuria	19.0% (12/63)
**Blood examination**	
White blood cell count, ×10^9^/L	14.9 (11.4–17.5)
Neutrophil percentage, %	78.6 (65.7–86.4)
Platelet, ×10^9^/L	298 (254–373)
Hemoglobin, g/L	113 (106–118)
C–reactive proteins, mg/L	84 (54–116)
Erythrocyte sediment rate, mm/h	63.5 (47.0–78.3)
Alanine transaminase, U/L	55.5 (31.8–158.8)
Aspartate transaminase, U/L	40.5 (28.8–83.0)
Albumin, g/L	38.0 (35.8–39.7)
Total bilirubin, μmol/L	8.9 (5.9–12.7)
Blood urea nitrogen, μmol/L	3.3 (2.7–4.1)
Creatinine, μmol/L	30.5 (25.0–37.3)
Serum sodium, mmol/L	136.0 (134.0–138.0)

*KD, Kawasaki disease; WBCs/HPF, white blood cells (WBCs)/high-power field (HPF); IVIG, intravenous immunoglobulin. The data are presented as the median with the 25th and 75th percentiles in square brackets for continuous variables and as the percentage for the categorical variables*.

### Comparison of the Clinical and Laboratory Data Between KD Patients With and Without Sterile Pyuria

As shown in Table 2, there were no significant differences in the sex ratio, average age, duration of fever before the initial IVIG administration, incidence of incomplete KD, typical clinical features, and the rate of delayed IVIG treatment between the groups (*p* > 0.05). The duration of antibiotic treatment was significantly shorter in patients with sterile pyuria than in the patients without sterile pyuria (3.9 ± 2.4 vs. 4.5 ± 1.6 days, *p* = 0.007). In addition, the rate of initial IVIG resistance (30.2 vs. 13.1%, *p* = 0.001), steroid treatment (19.0 vs. 6.7%, *p* = 0.002) as well as the rate of moderate/giant CAAs (6.1 vs. 0.9%, *p* = 0.002) were higher in the patients with sterile pyuria than in those without.

**Table 2:** The comparison of demographic, clinical characteristic and laboratory results of Kawasaki disease between patients with sterile pyuria and without.

**Clinical characteristic**	**Group A^**a**^ (*n =* 63)**	**Group B^**b**^ (*n =* 639)**	***P* value**
Sex, male, *n* (%)	38 (60.3)	362 (56.7)	0.597
Age, months	2.9 ± 2.0	2.6 ± 1.8	0.187
Incomplete Kawasaki disease, *n* (%)	19 (30.2)	244 (38.2)	0.223
**Typical clinical manifestations**			
Fever, *n* (%)	63 (100.0)	639 (100.0)	—
Rash, *n* (%)	56 (88.9)	501 (78.4)	0.051
Edema & erythema of the extremities, *n* (%)	36 (57.1)	357 (55.9)	0.895
Bilateral bulbar conjunctival injection, *n* (%)	60 (95.2)	585 (91.5)	0.466
Erythema of oral and pharyngeal mucosa, *n* (%)	59 (93.7)	575 (90.0)	0.502
Cervical lymphadenopathy, *n* (%)	29 (46.0)	277 (43.3)	0.692
**Treatment**			
The duration of antibiotic, days	3.9 ± 2.4	4.5 ± 1.6	0.007^*^
Fever duration before IVIG, days	5.2 ± 1.0	5.7 ± 1.4	0.015^*^
The delayed treatment of initial IVIG (>10 days)	1 (1.6)	13 (2.0)	1.000
Failure to respond to initial IVIG therapy, *n* (%)	19 (30.2)	84 (13.1)	0.001^*^
Steroid treatment, *n* (%)	12 (19.0)	43 (6.7)	0.002^*^
**Coronary artery classification**, ***n*** **(%)**			0.002^*^
Normal coronary artery	54 (85.7)	573 (89.7)	
CAD/small CAAs	5 (7.9)	60 (9.4)	
Moderate/giant CAAs	4 (6.3)	6 (0.9)	
**Blood examination features**			
White blood cell count, ×10^9^/L	14.7 ± 4.9	14.1 ± 5.1	0.289
Neutrophil percentage, %	75.9 ± 12.4	66.5 ± 14.9	<0.001^*^
Platelet, ×10^9^/L	302.8 ± 95.6	321.7 ± 120.9	0.227
Hemoglobin, g/L	112.6 ± 10.9	108.4 ± 11.9	0.007^*^
C–reactive proteins, mg/L	89.9 ± 48.5	81.6 ± 48.3	0.196
Erythrocyte sediment rate, mm/h	63.8 ± 25.4	65.9 ± 27.7	0.564
Alanine transaminase, U/L	117.0 ± 120.3	69.4 ± 87.7	0.003^*^
Aspartate transaminase, U/L	78.4 ± 101.4	53.3 ± 71.9	0.062
Albumin, g/L	37.6 ± 4.1	37.5 ± 4.6	0.857
Total bilirubin, μmol/L	13.1 ± 15.1	8.5 ± 10.3	0.022^*^
Blood urea nitrogen, μmol/L	3.7 ± 2.4	2.7 ± 1.0	0.003^*^
Creatinine, μmol/L	33.5 ± 14.5	27.9 ± 7.5	0.004^*^
Serum sodium, mmol/L	136.0 ± 2.8	136.6 ± 3.5	0.241

*IVIG, intravenous immunoglobulin; CAD, coronary artery dilation; CAAs, coronary artery aneurysms.*

a *Group A, KD patients had sterile pyuria.*

b *Group B, KD patients had no sterile pyuria. The data are presented as mean ± standard deviation (SD) for quantitative variables and as n/% for qualitative data as appropriate.*

* *P < 0.05*.

Before the initial IVIG treatment, the following parameters were significantly higher in patients with sterile pyuria than in those without, percentage of neutrophils, hemoglobin (Hb), C-reactive protein (CRP), alanine transaminases (ALT), total bilirubin (TBil.), blood urea nitrogen (BUN), and creatinine (Cr) (*p* < 0.005).

### The Predictive Value of Sterile Pyuria in the Initial IVIG Resistance in KD

A comparison of the clinical data between the initial IVIG-response group and the initial IVIG-resistant group is shown in Table 3 in detail. In terms of the sex proportion, age, duration of fever before the initial IVIG administration, the occurrence of incomplete KD, CALs, microscopic hematuria, and pyocytes, as well as the three typical clinical presentations of KD (edema and erythema of the extremities, bilateral bulbar conjunctival injection, and erythema of the oral and pharyngeal mucosa), there were no significant differences between the two groups. Compared to the patients from the initial IVIG-response group, patients from the initial IVIG-resistant group showed a higher incidence of rash, cervical lymphadenopathy, and sterile pyuria, with significantly higher levels of percentage of neutrophils, platelet count, CRP, ALT, TBil., BUN, and Cr, but significantly lower levels of albumin (ALB) and serum sodium (Na^+^) (*P* < 0.05).

**Table 3:** Comparison of clinical data between the groups of IVIG-response and IVIG-resistance in patients with Kawasaki disease.

	**IVIG–responsive**	**IVIG–resistance**	**Univariate analysis**	**Multivariate analysis**	
	**(*n =* 599)**	**(*n =* 103)**	***P* value**	**OR (95%CI)**	***p* value**
Male, *n* (%)	346 (57.8)	54 (52.4)	0.333		
Age, years	2.6 ± 1.8	3.0 ± 2.2	0.056		
**Clinical manifestations**					
Rash, *n* (%)	467 (78.0)	90 (87.4)	0.034^*^	1.403 (0.704–2.796)	0.335
Edema & erythema of the extremities, *n* (%)	334 (55.8)	59 (57.3)	0.830		
Bilateral bulbar conjunctive injection, *n* (%)	553 (92.3)	92 (89.3)	0.327		
Erythema of oral and pharyngeal mucosa, *n* (%)	538 (89.8)	96 (93.2)	0.367		
Cervical lymphadenopathy, *n* (%)	250 (41.7)	56 (54.4)	0.018^*^	1.266 (0.783–2.048)	0.336
Fever duration before IVIG, days	5.7 ± 1.4	5.4 ± 1.5	0.090		
Incomplete KD, *n* (%)	231 (38.6)	32 (31.1)	0.154		
Coronary artery lesions, *n* (%)	59 (9.8)	16 (15.5)	0.087		
**Urine analysis**					
Sterile pyuria, *n* (%)	44 (7.3)	19 (18.4)	0.001^*^	2.410 (1.215–4.781)	0.012^*^
Microscopic hematuria, *n* (%)	12 (2.0)	4 (3.9)	0.273		
Pyocytes, *n* (%)	17 (2.8)	1 (1.0)	0.496		
**Laboratory results before IVIG**					
White blood cell (WBC), ×10^9^/L	14.2 ± 5.1	13.5 ± 5.3	0.194		
Neutrophil percentage, %	66.3 ± 14.7	73.9 ± 14.6	<0.001^*^	1.474 (0.840–2.589)	0.176
Lymphocyte percentage, %	25.1 ± 12.2	17.5 ± 10.3	<0.001^*^		
Hemoglobin, g/L	109 ± 11	106 ± 14	0.066		
Platelet count, ×10^9^/L	327 ± 118	275 ± 114	<0.001^*^	1.438 (0.889–2.326)	0.139
C–reactive protein, mg/L	79.8 ± 47.8	97.1 ± 48.8	0.001^*^	1.407 (0.784–2.528)	0.253
Erythrocyte sedimentation rate, mm/h	65.1 ± 27.0	68.9 ± 30.1	0.199		
Aspartate aminotransferase, U/L	53.9 ± 76.8	65.1 ± 64.7	0.173		
Alanine aminotransferase, U/L	69.0 ± 87.9	101.4 ± 109.6	0.006^*^	0.994 (0.590–1.674)	0.981
Albumin, g/L	37.9 ± 4.4	35.2 ± 5.0	<0.001^*^	1.983 (1.160–3.388)	0.012^*^
Total bilirubin, μmol/L	7.8 ± 8.7	15.1 ± 18.1	<0.001^*^	3.353 (1.763–6.376)	<0.001^*^
Blood urea nitrogen, μmol/L	2.8 ± 1.1	3.2 ± 1.9	0.002^*^	0.912 (0.535–1.554)	0.734
Creatinine, μmol/L	27.9 ± 7.9	31.3 ± 10.7	<0.001^*^	1.062 (0.647–1.741)	0.812
Serum sodium, mmol/L	136.9 ± 3.3	134.6 ± 3.6	<0.001^*^	2.168 (1.323–3.553)	0.002^*^

*IVIG, Intravenous immunoglobulin; CAD, coronary artery dilation; CAAs, coronary artery aneurysms; KD: Kawasaki disease. The data are presented as mean ± standard deviation (SD) for quantitative variables and as n/% for qualitative data as appropriate.*

* *P < 0.05*.

These statistically significant variables from the univariate analysis mentioned above were included in the multivariate logistic regression analysis. It was identified that sterile pyuria, ALB ≤ 33 g/L, TBil ≥ 16.0 μmol/L, and Na^+^ ≤ 135.4 mmol/L were independent risk factors for the initial IVIG resistance. These results are presented in Table 4. The medical diagnostic test evaluation further predicted the initial IVIG resistance with sensitivity, specificity, positive predictive value (PPV), negative predictive value (NPV), and diagnostic accuracy using these variables (Table 5).

**Table 4:** A multivariate logistic regression model for intravenous immunoglobulin resistance in patients with Kawasaki disease.

	**β**	**SE**	**Wals**	***P* value**	**OR**	**95% CI**
Sterile pyuria	0.880	0.350	6.333	0.012	2.410	1.215–4.781
Rash	0.339	0.352	0.928	0.335	1.403	0.704–2.796
Cervical lymphadenopathy	0.236	0.245	0.924	0.336	1.266	0.783–2.048
Neutrophil percentage ≥ 81.3%	0.388	0.287	1.827	0.176	1.474	0.840–2.589
Platelet count ≤ 312 ×10^9^/L	0.363	0.245	2.187	0.139	1.438	0.889–2.326
C–reaction protein ≥ 57.4 mg/L	0.342	0.299	1.308	0.253	1.407	0.784–2.528
Albumin ≤ 33 g/L	0.685	0.273	6.271	0.012	1.983	1.160–3.388
Alanine aminotransferase ≥ 41 U/L	−0.006	0.266	0.001	0.981	0.994	0.590–1.674
Total Bilirubin ≥ 16 μmol/L	1.210	0.328	13.616	0.000	3.353	1.763–6.376
Serum sodium ≤ 135.4 mmol/L	0.774	0.252	9.440	0.002	2.168	1.323–3.553
Blood urea nitrogen ≥ 2.89 μmol/L	−0.092	0.272	0.115	0.734	0.912	0.535–1.554
Creatinine ≥ 26.0 μmol/L	0.060	0.252	0.056	0.812	1.062	0.647–1.741
Interpret	−1.731	0.452	14.699	0.000	0.177	

**Table 5:** The Ability of sterile pyuria, ALB, TBil, and Na^+^ cut-off values in IVIG resistance prediction.

	**AUC**	**SE**	**95%CI**	**Sensitivity**	**Specificity**	**PPV**	**NPV**	**Diagnostic** **accuracy**	***p* value**
Sterile pyuria (+)	0.559	0.020	0.521–0.596	0.185	0.927	0.302	0.869	0.818	<0.001
ALB ≤ 33 g/L	0.609	0.023	0.572–0.645	0.340	0.856	0.283	0.885	0.782	<0.001
TBil ≥ 16.0 μmol/L	0.598	0.025	0.560–0.634	0.280	0.938	0.431	0.886	0.843	<0.001
Na^+^ ≤ 135.4 mmol/L	0.645	0.026	0.609–0.681	0.590	0.700	0.248	0.911	0.684	<0.001

*ALB, albumin; TBil, total bilirubin; Na+, sodium; IVIG, intravenous immunoglobulin; NPV, negative predictive value; PPV, positive predictive value*.

### The Value of Sterile Pyuria in the Prediction of Repeated IVIG Resistance and CALs

The incidence of sterile pyuria did not differ between the repeated IVIG resistance group and repeated IVIG response group [23.7% (9/38) vs. 15.4% (10/65), *p* = 0.306] as well as between the regressed CALs group and the persistent CALs group [7.7% (4/52) vs. 21.7% (5/23), *p* = 0.122].

## Discussion

To the best of our knowledge, this is the largest study to systematically and prospectively elaborate on the clinical features of patients with sterile pyuria in KD. We also firstly evaluated the predictive value of sterile pyuria in patients with IVIG resistance, as well as the occurrence and persistence in KD. Our findings indicated that the incidence of sterile pyuria was relatively low among patients with KD, without predominance in any age group or sex. No significant delays in diagnosis or the initiation of IVIG infusion were observed in patients with sterile pyuria since these patients were more likely to present with the typical clinical features of KD. KD patients with sterile pyuria exhibited a more severe inflammatory burden and were more likely to develop an initial IVIG resistance and moderate/giant CAAs. These findings were of great clinical importance and significance since they suggested that KD patients with sterile pyuria should receive adjunctive therapies for primary treatment, such as corticosteroids, infliximab, and cytotoxic agents. However, the overall prognosis of KD patients with sterile pyuria seems to be satisfactory since sterile pyuria did not increase the risk of CALs persistence at 1-year follow-up from disease onset in patients with KD.

In terms of the incidence of sterile pyuria in patients with KD, 30–80% of patients with KD have previously been reported to present with sterile pyuria (6–9, 13–18). In contrast, in our study, the incidence of sterile pyuria was 9.0%, which was much lower. Several explanations can be proposed for the observed differences. First, pyuria is not always sterile in patients with KD. Wu et al. retrospectively reviewed the clinical and laboratory data of 75 patients with KD who underwent urinalysis and urine bacterial cultures and reported that 8 (10.7%) patients had bacterial pyuria (19). Similar findings were also observed in other studies. It was found that the KD patients could concurrently develop urinary tract infections due to *Escherichia coli* or *Klebsiella* regardless of the urinary tract abnormalities (20). In our cohort, bacterial pyuria and sterile pyuria related to other causes, such as renal tuberculosis, renal abscess, renal and urinary tract anomalies, and systemic lupus erythematosus, were found in 10 and 13 KD patients, respectively, which were finally excluded from our study. In contrast, the definition of sterile pyuria, inclusion criteria, and exclusion criteria were unclear and even lacking in most previous studies (6–9, 13–18), which might have resulted in an overestimation of the incidence of sterile pyuria since patients with bacterial pyuria and sterile pyuria due to other causes might also be included. Second, all the previous studies were limited by their retrospective design and small sample size (6–9, 13–17). The sample size in most studies was less than 50 (6, 8, 16), and the maximum sample size of 145 was present in the study by Liu et al. (13). Therefore, due to the above limitations, it was quite difficult to reach a definitive and accurate incidence of sterile pyuria in KD from previous studies. In contrast, due to the sufficient number of patients and the prospective approach, the findings in our report might be more convincing and conclusive.

With respect to the age distribution of sterile pyuria in patients with KD, data are limited, and inconsistent conclusions are drawn. Wirojanan et al. (9) and Liu et al. (13) reported that pyuria is more common in patients less than 1 year of age. However, the results from the studies by Choi et al. (7) and Barone et al. (16) documented that there were no significant differences in the age distribution of patients with pyuria in KD. As aforementioned, the variations in the definition of sterile pyuria, inclusion criteria, and exclusion criteria among these studies might contribute to the differences. As for the origin of sterile pyuria in patients with KD, several possible underlying mechanisms have been hypothesized. Amano et al. studied the systemic pathological alterations in patients with KD and found patients with proteinuria and pyuria in KD had urinary system inflammation (21). Melish et al. (17) performed bladder taps on 4 KD patients with pyuria and compared them with the voided urine specimens, and reported the bladder urine to be free of WBCs, suggesting that the urethral inflammation was the source of pyuria in patients with KD. However, a recent study by Watanabe et al. found that 5 of 10 KD patients with sterile pyuria in the voided urine samples also had leukocytes in the bladder urine (6), suggesting that some patients with KD develop sterile pyuria that originates from the inflammation in the kidneys as a result of mild and sub-clinical renal parenchymal injuries. This hypothesis was further confirmed by the imaging studies and/or urinary cytokine analysis. Our findings also supported that sterile pyuria might originate, in part, from the kidney, since 19% of patients with KD with sterile pyuria had microscopic hematuria with dysmorphic RBCs. However, the exact mechanisms related to the development of sterile pyuria in KD warrant further clarification.

As expected, our data indicated that patients with sterile pyuria had a higher incidence of initial IVIG resistance. This finding could be explained by the increased severity of the inflammatory burden in patients with sterile pyuria. It was widely recognized that an increase in the inflammatory burden was associated with IVIG resistance (1). In 1980, Amano et al. found that patients with proteinuria and pyuria in KD may exhibit focal interstitial nephritis, cystitis, and prostatitis (21). The study by Ohta et al. showed that the levels of urinary interleukin-6, N-acetyl-beta-D-glucosaminidase, and β2-microglobulin increased during the acute phase of KD (22), which proved the presence of an inflammatory process within the renal parenchyma. Moreover, Kawamura et al. found the activity of urinary lactate dehydrogenase originating from the urinary tract was significantly associated with IVIG resistance in KD (23). In line with previous findings, both our study and the study by Choi et al. (7) have demonstrated that the inflammatory indicators such as neutrophils' percentage, CRP, ESR, ALT, TBil, BUN, and Cr were significantly higher in KD patients with sterile pyuria. These evidences suggested that the inflammatory burden was increased in patients with sterile pyuria. Although sterile pyuria was not an ideal parameter for the initial IVIG resistance prediction because of its low sensitivity, we could distinguish those patients without an initial IVIG resistance by applying this indicator because of its extremely high specificity (92.7%).

Lastly, the prediction of CALs is equally of clinical importance and significance as IVIG resistance since KD patients with CALs may develop thrombosis or stenotic lesions and are at risk of myocardial infarction, sudden death, and congestive heart failure in the future. The findings of Choi et al. and Sepahi et al. (7, 8) suggested that the presence of sterile pyuria did not increase the risk of CALs. However, these studies were limited by the small sample size (*n* = 47 and *n* = 133, respectively) and retrospective nature. With a large contemporary cohort of KD patients, our study firstly found that the rate of moderate/giant CAAs was significantly higher in KD patients with sterile pyuria, which could be explained by the higher inflammatory reaction in these patients.

This study must be viewed in light of some potential limitations. First, our study might have been affected by a selection bias since it was single-center research, and our institution is the largest pediatric medical center in Southwest China. Second, the present study was a prospective cohort study with strict inclusion and exclusion criteria. The findings of this study are, therefore, applicable only to Chinese patients with KD receiving standardized IVIG treatment (2 g/kg) within 10 days of fever onset.

## Conclusion

Our findings indicated that the incidence of sterile pyuria is low among patients with KD, without any differences in the different age groups and sex. Sterile pyuria in KD might partly originate from the inflammation in the kidneys. Patients with KD with sterile pyuria exhibited a more severe inflammatory burden and were more likely to develop an initial IVIG resistance and moderate/giant CAAs, suggesting these patients should receive adjunctive therapies for primary treatment, such as corticosteroids, infliximab, and cytotoxic agents. However, the overall prognosis of KD patients with sterile pyuria was satisfactory.

## Data Availability Statement

The original contributions presented in the study are included in the article/supplementary material, further inquiries can be directed to the corresponding author/s.

## Ethics Statement

The studies involving human participants were reviewed and approved by The University Ethics Committee on Human Subjects at Sichuan University approved the study. Written informed consent to participate in this study was provided by the participants' legal guardian/next of kin.

## Author Contributions

XL drafted the manuscript, contributed to the data collection, interpreted the statistical analysis, approved financial support, and as well as approved the final manuscript as submitted. LW drafted the manuscript, contributed to the data collection, and approved the final manuscript as submitted. SS provided Tables 1, 2, contributed to the data collection, and approved the final manuscript as submitted. NZ contributed to the data collection, interpreted the statistical analysis, and approved the final manuscript as submitted. MW and LL contributed to the data collection and approved the final manuscript as submitted. YH provided major treatment to these patients while admitted, contributed to the study design, approved financial support, and as well as approved the final manuscript as submitted. KZ provided Tables 3–5, contributed to the study design, and as well as approved the final manuscript as submitted. HW and CW conceived conception and designed the study, contributed to the data collection, approved financial support, and as well as approved the final manuscript as submitted. LY contributed to the study design, approved financial support, and as well as approved the final manuscript as submitted. All authors contributed to the article and approved the submitted version.

## Funding

This study was supported by Science Technology Support Plan Projects in Sichuan province (Grant No. 22DYF2160, 2020YJ0234, and 2019YFS0241), the National Natural Science Foundation of China (Grant No. 81971457 and 82070324).

## Conflict of Interest

The authors declare that the research was conducted in the absence of any commercial or financial relationships that could be construed as a potential conflict of interest.

## Publisher's Note

All claims expressed in this article are solely those of the authors and do not necessarily represent those of their affiliated organizations, or those of the publisher, the editors and the reviewers. Any product that may be evaluated in this article, or claim that may be made by its manufacturer, is not guaranteed or endorsed by the publisher.

## Abbreviations

AHA: American Heart Association
ALB: Albumin
ALT: Alanine aminotransferase
AST: Aspartate aminotransferase
AUC: Area under the curve
BUN: Blood urea nitrogen
BSA: body surface area
Cr: creatinine
CBC: Complete blood count
CRP: C-reactive protein
CAD: coronary artery dilation
CAA: coronary artery aneurysms
CALs: Coronary artery lesions
ESR: Erythrocyte sedimentation rate
Hb: hemoglobin
IVIG: Intravenous immunoglobulin
KD: Kawasaki disease
NPV: Negative predictive value
Na+: Sodium
ORs: Odds ratios
PPV: Positive predictive value
ROC: Receiver operating characteristic
SD: Standard deviation
TBil: Total bilirubin
WBC: White blood cell.
